# The effects of wildfire severity and pyrodiversity on bat occupancy and diversity in fire-suppressed forests

**DOI:** 10.1038/s41598-019-52875-2

**Published:** 2019-12-05

**Authors:** Z. L. Steel, B. Campos, W. F. Frick, R. Burnett, H. D. Safford

**Affiliations:** 1 Department of Environmental Science and Policy, University of California, One Shields Avenue, Davis, CA 95616 USA; 2 Point Blue Conservation Science, Petaluma, CA 94954 USA; 3 Bat Conservation International, Austin, Texas 78746 USA; 4 Department of Ecology and Evolutionary Biology, University of California Santa Cruz, California, 95060 USA; 5 United States Department of Agriculture, Forest Service, Pacific Southwest Region, Vallejo, CA 94592 USA

**Keywords:** Biodiversity, Community ecology, Conservation biology, Fire ecology, Forest ecology

## Abstract

Wildfire is an important ecological process that influences species’ occurrence and biodiversity generally. Its effect on bats is understudied, creating challenges for habitat management and species conservation as threats to the taxa worsen globally and within fire-prone ecosystems. We conducted acoustic surveys of wildfire areas during 2014–2017 in conifer forests of California’s Sierra Nevada Mountains. We tested effects of burn severity and its variation, or pyrodiversity, on occupancy and diversity for the 17-species bat community while accounting for imperfect detection. Occupancy rates increased with severity for at least 6 species and with pyrodiversity for at least 3. Two other species responded negatively to pyrodiversity. Individual species models predicted maximum occupancy rates across burn severity levels but only one species occurred most often in undisturbed areas. Species richness increased from approximately 8 species in unburned forests to 11 in pyrodiverse areas with moderate- to high-severity. Greater accessibility of foraging habitats, as well as increased habitat heterogeneity may explain positive responses to wildfire. Many bat species appear well adapted to wildfire, while a century of fire suppression and forest densification likely reduced habitat quality for the community generally. Relative to other taxa, bats may be somewhat resilient to increases in fire severity and size; trends which are expected to continue with accelerating climate change.

## Introduction

Wildfire is an important driver of the distribution and structure of ecosystems globally^1,2^. In the frequently burned forests of the western United States, a single wildfire often has disparate effects on habitat ranging from maintaining mature conifer forests to complete removal of aboveground live vegetation^3,4^. Habitat heterogeneity created by fire, often referred to as pyrodiversity, may be important for promoting biodiversity generally in fire-dependent systems^5^. In particular, fire influences bat foraging and roosting resources provided by montane forest ecosystems and may be an important driver of habitat quality^6^. However, the effects of wildfire on bat occurrence and diversity within forest landscapes are understudied, creating challenges for bat conservation and management in fire-prone ecosystems^7,8^.

How wildfires alter local habitat structure, landscape composition of vegetation physiognomies, and biodiversity depends on the severity, and spatial pattern of individual and successive disturbances^3,4^. Prior to euromerican settlement, frequent wildfire in the dry forests of California’s Sierra Nevada Mountains burned primarily at low- and moderate-severities^4,9,10^, creating a mix of vegetation types and successional stages^11^. This pyrodiversity, may have helped maintain biodiversity in the region historically^5^. Over the past century, much of the region has been managed under a policy of fire suppression^4^, which together with past logging practices, has led to broad changes in Sierra Nevada forest ecosystems including forest densification and declining landscape heterogeneity^12^. With the interaction of forest fuel accumulation and a warming climate, fires that escape suppression efforts are burning with increasingly high-severity^13,14^, creating larger and more homogenous high-severity patches^15^. Such broad shifts in the regional fire regime have important implications for biodiversity adapted to historical fire patterns, and raise questions about the maintenance of biodiversity of fire-sensitive taxa^15,16^.

Bats are important components of wildlife communities globally, making up approximately a fifth of mammalian diversity^17^. They also provide valuable ecosystem services and economic benefits to society, including the control of agricultural and forest pests^18^. At the same time, bats face myriad threats including habitat loss, climate change, wind energy development, and emerging diseases^19–22^. In particular, the fungus *Pseudogymnoascus destructans* that causes white-nose syndrome in bats is expanding into western habitats from the eastern Unites States, where it has devastated bat populations in recent years^20^. Further, a warming climate is increasing the frequency and severity of droughts^23^, which can directly result in large bat mortality events^22^, and indirectly affect bat habitat quality through interactions with ecosystem processes such as wildfire^24^.

Fires alter bat foraging habitat structure by reducing clutter in the form of tree and understory vegetation density. The ability of insectivorous bats to navigate and capture prey in cluttered environments depends in part on echolocation call characteristics and body morphology^25^. Species with short duration calls and small body size relative to wing area are tolerant of cluttered environments, whereas longer duration, low-frequency calls, and relatively large bodied species are more associated with open environments^6,25^. Such variation in habitat associations leads to the expectation that wildfire severity would have idiosyncratic effects on occurrence among species, and that more pyrodiverse landscapes would promote higher community diversity.

To evaluate the effects of burn severity and pyrodiversity on bats in Sierra Nevada forests, we conducted acoustic surveys within and near three wildfire areas across a four-year period in the Sierra Nevada Mountains of California. We used Bayesian hierarchical modeling that accounts for rates of imperfect detection and recordings from 1274 survey nights, constituting 205 survey periods to address two primary questions: (1) Does site occupancy of bat species differ across gradients of mean burn severity, and with variation in burn severity? (2) Do these severity gradients affect alpha and beta diversity of the forest bat community?

## Methods

### Study region & survey design

**Figure 1:**
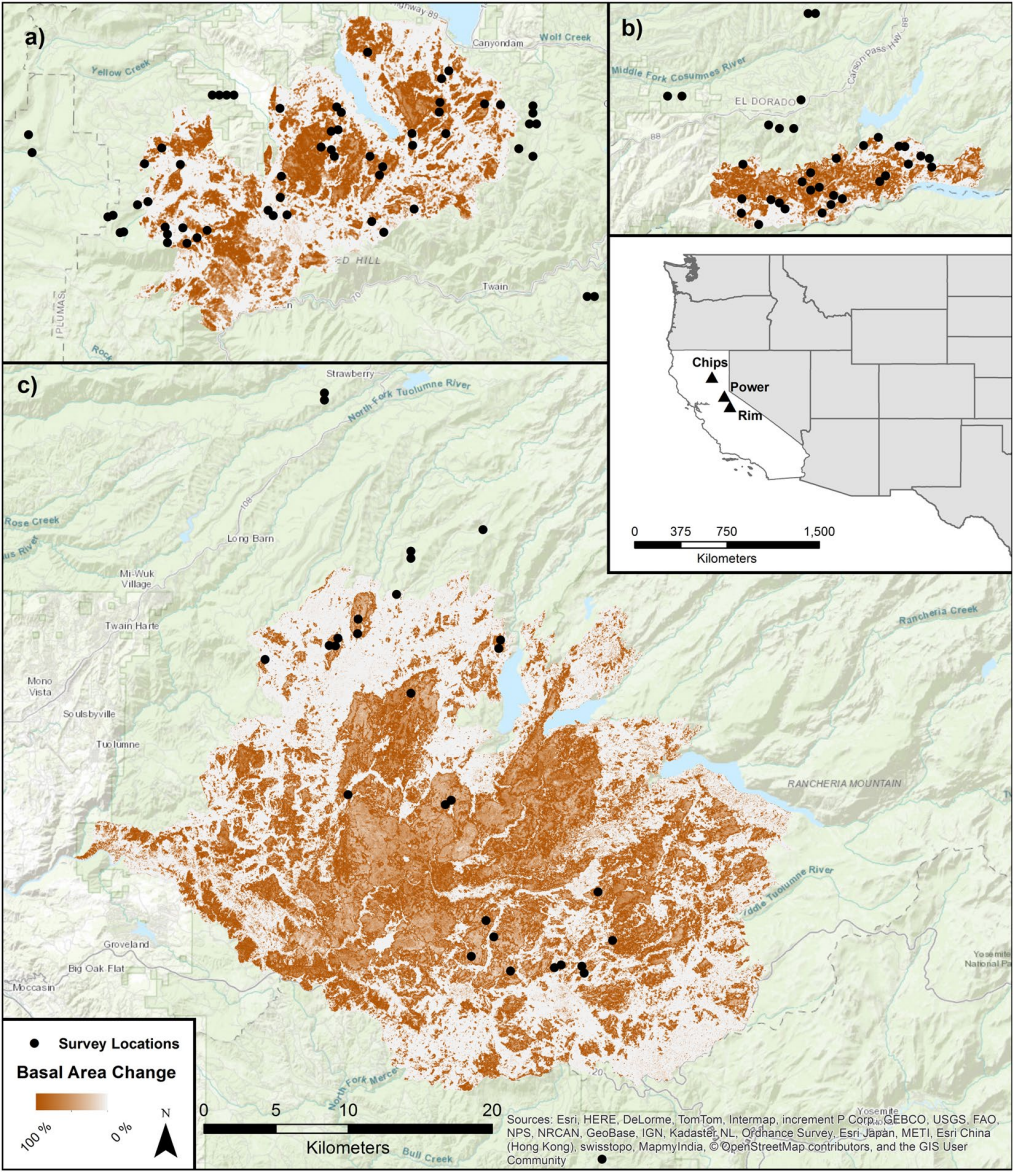
Percent change in forest basal area from the (**a**) Chips fire, (**b**) Power fire, and (**c**) Rim fire, along with survey locations within and outside fire perimeters. All fires are mapped at the same spatial scale. An insert of California and the western United States is provided for reference. Maps were created using ArcGIS software and topographic basemap^53^.

Bat surveys were conducted during the spring/summer season of 2014–2017 within three areas burned by large wildfires in the mid-montane zone of the Sierra Nevada Mountains of California. The wildfires included the 2004 Power fire in Eldorado National Forest, the 2012 Chips fire in Lassen and Plumas National Forests, and the 2013 Rim fire in Stanislaus National Forest (Fig. 1). The region is characterized by yellow pine and mixed-conifer forests interspersed with chaparral, meadows, and riparian areas^26^. Pre-fire and reference forests are dominated by *Pinus ponderosa, Abies concolor*, and *Pseudotsuga menziesii*, transitioning to *A. magnifica*, and *Pinus jeffreyi* at higher elevations. Hardwood species, especially *Quercus* spp., *Arctostaphylos* spp., and *Ceanothus* spp. are also found in conifer stands or as the dominant vegetation in recently burned areas^4^. Survey locations were limited to National Forest lands and areas of conifer forests prior to wildfires of interest.

Bat survey locations were selected using a stratified random sampling protocol where the population of potential survey locations was limited to established avian point count stations as part of ongoing regional and post-fire monitoring programs^27,28^. Co-locating survey efforts allowed for more efficient use of researcher time, and a multi-taxa understanding of wildlife response to wildfire effects. Points were spaced a minimum of 500 m apart (mean minimum distance = 1193 m), with a single exception where two points were located 250 m apart. The following four strata were used in site selection: (1) unburned reference points outside of fire perimeters, (2) low-severity, (3) moderate-severity, and (4) high-severity. Potential points were categorized according to remotely sensed severity classes as defined by Miller and Thode^29^. Our low-severity category also included Miller and Thode’s “unchanged” areas within a burn perimeter. These areas are included in the lower-severity rather than the unburned category because they either burned at very low-severity or represent small islands of unburned vegetation within a matrix of higher severity. All unburned points were located within the same forest types and elevation band as the burned survey locations. Surveys were excluded from analysis if a site had previously experienced post-fire management (i.e. salvage logging and reforestation activities) across a least 10% of the area within 50 m of the sample point. Post-fire management actions and their spatial extent were determined using the Forest Service Activity Tracking System database, and vegetation surveys. Severity and activity databases can be found at https://www.fs.usda.gov/main/r5/landmanagement/gis.

Bat surveys were conducted using acoustic detectors (SM3BAT - Wildlife Acoustics inc.) coupled with ultrasonic microphones that provide full spectrum recordings of bat echolocation calls (SM3/SMM_U1 - Wildlife Acoustics inc.). Each microphone was tested for potential loss of sensitivity before and after each field season. Microphones were elevated approximately 2.5 m above the ground and were located away from sources of clutter (e.g. branches), and sound-reflective surfaces that might distort recordings. Microphones were oriented toward open areas relative to the habitat being sampled to maximize the likelihood of recording a passing bat. Detectors were deployed for approximately two-week survey periods during a season, recording on alternate nights from 30 minutes prior to sunset to 30 minutes following sunrise. Nights when bats were not surveyed were used to sample for owl calls in a complementary study. The number of survey nights per survey period ranged from 1 to 17 with a median of 6, depending on battery life and equipment function. During each survey period, detectors were placed across the severity gradient - in each of the four strata described above. We sampled 122 unique sample locations over the four years. Surveys were conducted between May and September, which includes the energetically-demanding maternity period when females give birth and raise young. 52% of sites were surveyed during a single year, 31% during two years, 15% during three years, and 2% during all four years. In total, bat occurrence data were collected from 205 survey periods (site and year combinations) including a total of 1274 survey nights (Table 1).

**Table 1:** Acoustic monitoring effort for 2014–2017 across three burned areas and surrounding unburned forest.

Wildfire Area*	Burn Year	Elevational Range (m)	Survey Years	Survey Periods	Survey Nights
Power fire	2004	1230–1979	2014–2017	79	559
Chips fire	2012	1177–2055	2015–2016	69	357
Rim fire	2013	1169–2142	2014–2016	57	358
All				205	1274

*Includes unburned sites in Lassen, Plumas, Eldorado and Stanislaus National Forests.

### Environmental data

For analysis of severity effects, we converted Relativized differenced Normalized Burn Ratio values derived from LANDSAT-TM to estimates of percent basal area mortality according to Miller and Quayle^30^. All area outside of burn perimeters are considered to be unchanged with 0% basal area mortality. Bats are highly vagile animals, with foraging area varying widely among and even within species^6^. Due to this variation, the scale at which species respond to wildfire may also differ. To account for the influence of scale on habitat selection, we calculated the mean and standard deviation of basal area change for each point using buffers around sites defined by radii of 50, 100, 250 and 500 m.

Elevation of each survey location was extracted from a 30 m resolution digital elevation model (nationalmap.gov/elevation.html). We calculated distance to perennial water (i.e. streams and lakes) from each sample location using the National Hydrography Dataset (http://nhd.usgs.gov/). We estimated canopy cover within a 15 m radius of the microphone for each survey period using a Releve’ method adapted from the California Department of Fish and Wildlife’s standard protocol. We measured ambient noise rate and mean temperature for each survey night. Ambient noise rates were calculated as the ratio of noise-recordings (i.e. instances where a recording was initiated by non-bat sounds) to total recordings per night. We recorded temperatures every minute during sampling with the internal SM3BAT thermometer and calculated a nightly mean value for analysis.

### Species classification

We classified recorded bat pulses to the species-level when possible using Sonobat version 3.2.1 and the western regional library, which is populated with recordings from all 17 species assessed (www.sonobat.com). Classifications were made using SonoBat’s autofilter setting, acceptable call quality threshold of 0.80, sequence decision threshold of 0.90 and a maximum of 8 calls considered per file. Sonobat software assigns a likelihood of presence and a nightly corrected count of each species in a given night. Corrected counts are a conservative estimate of the number of recordings for each species as many low quality or ambiguous recordings are not classified to species. Specifically, 62% of recordings containing a bat pass received an “unknown” species classification. The automated classification process is intentionally conservative to reduce the likelihood of problematic false presences, but doing so increases the rate of false absences, which are more easily accounted for statistically^31^. A species was considered observed during a given night if at least one corrected count was tallied by the automated classifier, and unobserved/not detected otherwise.

In addition to challenges in classification of recorded passes, a species may be present but not recorded. These two sources of false-absences result in imperfect detection of a species in a given night. Imperfect detection is a common problem when surveying mobile and inconspicuous wildlife^32^, and is particularly problematic for some bat species^31^. Within a given habitat and atmospheric conditions, detection distances can vary widely among species based on intensity and frequency of echolocation calls. In order to provide unbiased estimates of occurrence and effects of environmental covariates, we employed occupancy models to estimate detection probabilities as well as occurrence rates^31,32^.

### Statistical analysis

We consider each site and year combination as a survey period (*i*), and each survey night as a temporal replicate (*j*). The community is assumed to be closed during each season (May-September), allowing for formal estimation of rates of detection and true occupancy. Observation data are binary with *y*_*i,j*_ = 1 when a species is observed during a survey period *i* and night *j*, or *y*_*i,j*_ = 0 when a species was not recorded, or a classification could not be confirmed. Because detection is imperfect, we utilize the following occupancy modeling framework: ${y}_{i,j}=Bernoulli({p}_{i,j}\ast {z}_{i})$ where *p*_*i,j*_ is the probability of detection given a site is occupied (i.e. *z*_*i*_ = 1). A species’ true occurrence is modeled as *z*_*i*_ = *Bernoulli*(*Ψ*_*i*_) where *Ψ*_*i*_ is the probability of occurrence, and *z*_*i*_ is a binary latent variable of a species’ true occurrence state. We assumed detection and occurrence probabilities vary by survey period, and are functions of habitat and survey characteristics. The detection process is modeled using the following function:

$$\begin{array}{rr}logit({p}_{ij})= & {\alpha }_{0}+{\alpha }_{1}\ast da{y}_{ij}+{\alpha }_{2}\ast da{y}_{ij}^{2}+{\alpha }_{3}\ast canop{y}_{i}+\\  & {\alpha }_{4}\ast nois{e}_{ij}+{\alpha }_{5}\ast temperatur{e}_{ij}+{\alpha }_{6}\ast sm{m}_{i}\end{array}$$

where *day*_*i,j*_ and $da{y}_{ij}^{2}$, *canopy*_*i*_, *noise*_*ij*_, *temperature*_*ij*_ are continuous predictors, and *smm*_*i*_ is an indicator of whether a SMM_U1 microphone was used. The two microphone models differ only in their housing, but our SMM_U1 units were purchased one year later than the SM3-U1 units, so potentially maintained higher sensitivity. The occurrence process is modeled using the following function:

$$\begin{array}{rcl}logit({\psi }_{i}) & = & {\beta }_{0}+\beta site[i]+\beta area[i]+\\  &  & {\beta }_{1}\ast elevatio{n}_{i}+{\beta }_{2}\ast water.distanc{e}_{i}+\\  &  & {\beta }_{3}\ast severit{y}_{i}+{\beta }_{4}\ast severit{y}_{i}^{2}+{\beta }_{5}\ast pyrodiversit{y}_{i}\end{array}$$

where *βsite*[*i*] and *βarea*[i] are random intercepts for site and wildfire area ID, respectively. For each site, *elevation*_*i*_, *water.distance*_*i*_, *severity*_*i*_, $severit{y}_{i}^{2}$, and *pyrodiversity*_*i*_ are continuous predictors of occupancy. Severity and its quadratic are defined as percent forest basal area mortality. Similar to Tingley *et al*.^33^ we define pyrodiversity as the heterogeneity of burn severity around a survey point. However, instead of using the standard deviation of burn severity (*sev*_*sd*_*i*_), which is strongly related to mean severity (Figure S1), we adjust for mean severity using residual regression^34^. Specifically, *pyrodiversity*_*i*_ is the residual value from the linear model $$\begin{array}{c}sev\_s{d}_{i}=se{v}_{i}+se{v}_{i}^{2}\end{array}$$, and should be interpreted as variation in burn severity corrected for mean severity. We allow intercepts of *ψ* to vary by site and wildfire area ID to help account for spatial clustering of points, and to make explicit the assumption that occupancy at a site is correlated among years. All continuous predictors were standardized with a mean of zero and standard deviation of one. Thus, modeled intercepts of detection (*α*_0_) and occupancy (*β*_0_) are equivalent to mean estimates on the logit scale for each species, respectively.

For each species, the above model was run four times with mean severity and pyrodiversity calculated at different spatial scales, as defined by radii of 50, 100, 250, and 500 m from a site. The four models are compared for relative out-of-sample predictive power using the leave-one-out information criteria ^LOOIC;^^35^, and the best candidate model was selected according to the lowest LOOIC value (Table S1 and Figure S2). Models were estimated using Hamiltonian Monti Carlo sampling in Stan and program R via the rstan package^36,37^. We specified regularizing priors on site random intercepts and weakly regularizing priors on all other parameters to prevent model over fitting^38^. Models were run with 3 chains, each for 2000 samples with a warmup of 1000. Traceplots and R-hat values were assessed for proper mixing and model convergence. Full model specification and data can be found in the supplementary material.

We used species richness as a measure of alpha diversity. Richness was calculated as ${\sum }_{s=1}^{S}{\psi }_{s}$ where *ψ*_*s*_ is a function of burn severity (both linear and quadratic terms) and each of three levels of pyrodiversity (i.e. −2, 0 and 2 standard deviations from the mean), with other covariates held at their mean values. We calculated beta diversity using the jaccard dissimilarity index for each pair-wise combination of points, and each sample of model posteriors. To incorporate estimates of detectability in diversity estimates, occupancy models were used to predict occurrence matrices at each point. Jaccard coefficients were correlated with severity distance (difference in basal area loss) for each point combination, and mean values were compared between four severity groups: unchanged (0% basal area mortality), low (1–25%), moderate (25–75%), and high (>75%). For analysis of community-diversity we used mean burn severity calculated with 250 m radii because this represented the median selected scale among species (Table S1). We used the full posterior distribution of each species model to propagate uncertainty to the community level for both alpha and beta diversity calculations.

## Results

**Figure 2:**
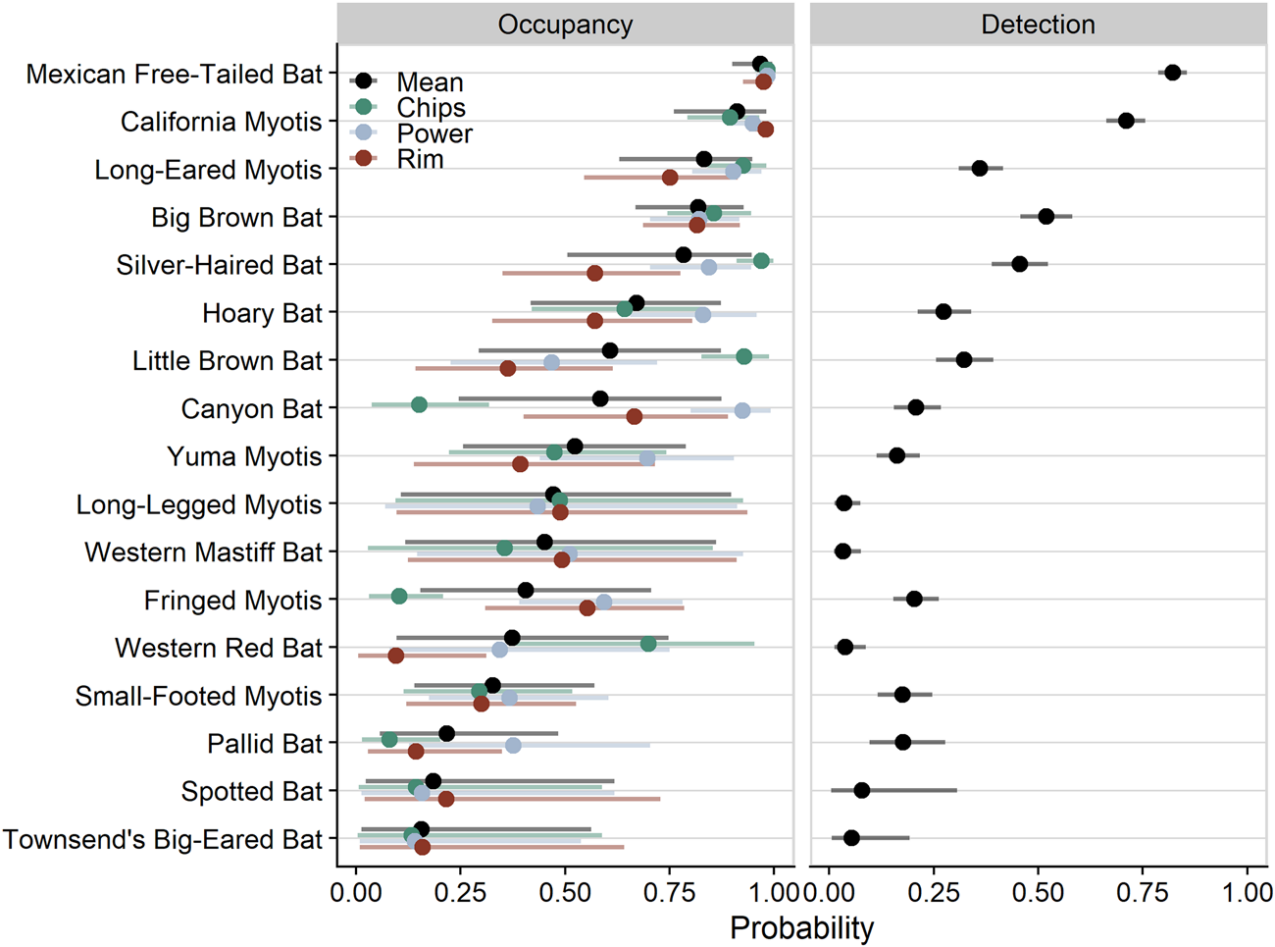
Estimated rates of occupancy and detection for each species. Mean estimates are displayed as points with 90% credible intervals displayed as bars. Varying occupancy rates for each wildfire area are also shown.

Modeled occupancy and detection rates varied greatly among the 17 bat species that occur in the Sierra Nevada of California. When model covariates are kept at their mean values, Mexican free-tailed bat (*Tadarida brasiliensis*) was predicted to be nearly ubiquitous with a mean occupancy rate of 97%, followed by California myotis (*Myotis californicus*) at 91%, long-eared myotis (*M. evotis*) at 83%, and big brown bat (*Eptesicus fuscus*) at 82%. The least common species were Townsend’s big-eared bat (*Corynorhinus townsendii*) with mean occupancy rates of 15% and spotted bat (*Euderma maculatum*) at 19% (Fig. 2). For some species, estimated occupancy rates varied widely among fires, especially for little brown bat (*M. lucifugus*), canyon bat (*Parastrellus hesperus*), fringed myotis (*M. thysanodes*), and western red bat (*Lasiurus blossevillii*). We did not explicitly test an effect of time since fire on occupancy, but five species are estimated to occur more often (with varying certainty) in the older 2004 Power Fire, despite its intermediate latitude relative to the two other fires. No single fire had consistently greater occupancy rates across species (Fig. 2).

The most easily detected species include Mexican free-tailed bat and California myotis, which are expected to be detected during more than 70% of survey nights given a site is occupied. Conversely, 10 species are expected to be observed during less than 25% of survey nights at an occupied site (Fig. 2). The most common species are often detected the most easily with some exceptions. For example, long-legged myotis (*M. volans*) is a relatively common species in the central Sierra (Frick *personal observation*), but their calls can be difficult to distinguish from little brown bat. This likely resulted in many false absences in the data, with consequently low detection rates and high uncertainty in occupancy rates (Fig. 2).

### Species occupancy effects

**Figure 3:**
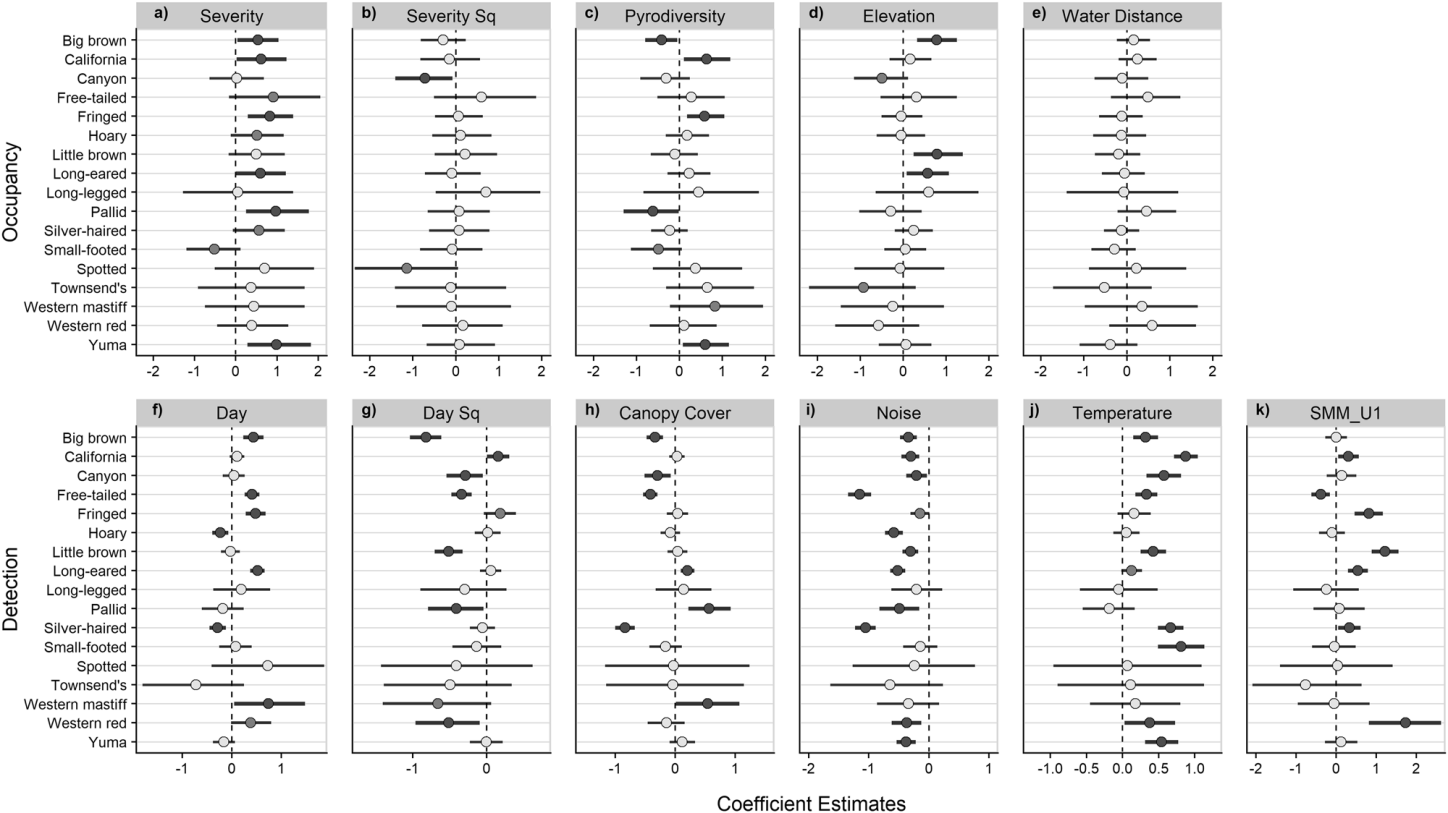
Dot plots of standardized coefficient estimates for occupancy and detectability. Points represent mean estimates and bars represent 90% credible intervals. Estimates where the probability of a positive or negative effect is greater than 90% and 95% are emphasized by light and dark grey shading, respectively. Full common and scientific names can be found in Table S1. Tabulated coefficient estimates can be found in Table S2.

**Figure 4:**
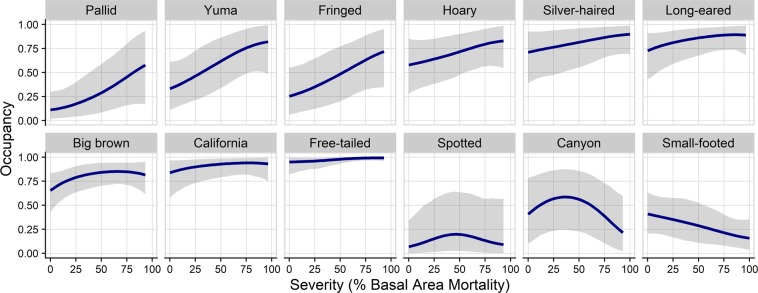
Marginal effect of burn severity (percent basal area mortality) on bat occupancy, arranged by effect size. Mean predictions and 90% credible intervals are shown. Species are excluded if both the linear and quadratic effects of severity are highly uncertain (i.e. where the probability of a positive and negative effect is <90%). Full common and scientific names, and the scale of final species models can be found in Table S1.

**Figure 5:**
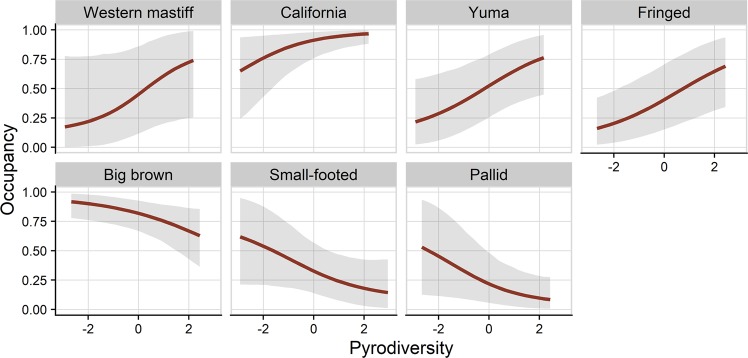
Marginal effect of pyrodiversity (standardized variation in burn severity) on bat occupancy, arranged by effect size. Mean predictions and 90% credible intervals are shown. Species are excluded if the effect of pyrodiversity is highly uncertain (i.e. where the probability of a positive and negative effect is <90%). Full common and scientific names, and the scale of final species models can be found in Table S1. Predictions are limited to the range of pyrodiversity sampled at each respective scale.

Occupancy was positively affected by burn severity for 6 species with at least a 95% probability (Fig. 3a). Of these species, models predict occupancy to maximize between 65% and 87% of basal area mortality for California myotis, long-eared myotis, and big brown bat. Pallid bat (*Antrozous pallidus*), fringed myotis and Yuma myotis (*M. yumanensis*) are predicted to occur most often in areas with 100% basal area mortality (Fig. 4). Silver-haired bat (*Lasionycteris noctivagans*), Mexican free-tailed bat, and Hoary bat (*Lasiurus cinereus*) are also likely positively associated with burn severity, but with less model certainty (>90% probability). Small-footed bat (*M. ciliolabrum)* is the only species that showed a potential negative effect of mean burn severity (92% probability) and is expected to occur most often in unburned forests. The effect of burn severity is convex for canyon bat (>97% probability) and likely convex for spotted bat (94% probability) with the maximum occupancy rate predicted to occur at 40% and 45% basal area mortality, respectively, and lower rates predicted at severity extremes (Figs 3b and 4). Occupancy by fringed myotis, California mytis and Yuma myotis is positively affected by pyrodiversity (>96% probability) indicating higher occurrence rates with high variation in basal area mortality. Western mastiff bat (*Eumops perotis*) is also likely positively associated with pyrodiverse areas but with greater uncertainty (>91% probability). Conversely, big brown bat and pallid bat are negatively associated with pyrodiversity (>95% probability), suggesting they may prefer more homogenous habitats. Small-footed myotis shows a likely negative associated with pyrodiversity but with more uncertainty (>93% probability; Figs 3c and 5). Bat occupancy increased with elevation for big brown bat, little brown bat, and long-eared myotis (Fig. 3d). No species showed clear effects of distance to water (Fig. 3e).

### Species detectability effects

Detection covariates were notably influential for many species. Long-eared myotis, Mexican free-tailed bat, big brown bat, and fringed myotis were detected more consistently later in the sampling season (>99% probability), whereas silver-haired bat, and hoary bat showed the opposite trend (>99% probability; Fig. 3f). Big brown bat, Mexican free-tailed bat, little brown bat, canyon bat, western red bat, and pallid bat models estimate negative quadratic terms for day indicating the likelihood of recording the species peaks at some mid-point in the season, with California myotis showing the opposite (>95% probability; Fig. 3g). Mexican free-tailed bat, silver-haired bat, big brown bat, and canyon bat were detected less often with increasing canopy cover (>98% probability), consistent with the idea that vegetation between foraging bats and microphones would inhibit recording of echolocation calls. However, long-eared myotis pallid bat, and western mastiff bat were detected more often with increasing canopy cover (>95% probability; Fig. 3h), which may indicate the species are changing their calls in a way that increases detectability (e.g. by making their calls more easily distinguishable from other species). For example, pallid bat often forages using auditory cues of prey only^39^, and may increase the frequency of echolocations to navigate in more cluttered environments. Effects of noise and temperature were largely consistent across species with 11 expected to have lower rates of detection during noisy nights, and at least 9 species expected to have higher rates of detection during warmer nights (>95% probability; Fig. 3I,j). Long-eared myotis, little brown bat, fringed myotis, western red bat, silver-haired bat, and California myotis were detected at higher rates when the newer SMM_U1 microphone was used, whereas Mexican free-tailed bat was detected less often (>99% probability; Fig. 3g).

### Community diversity

**Figure 6:**
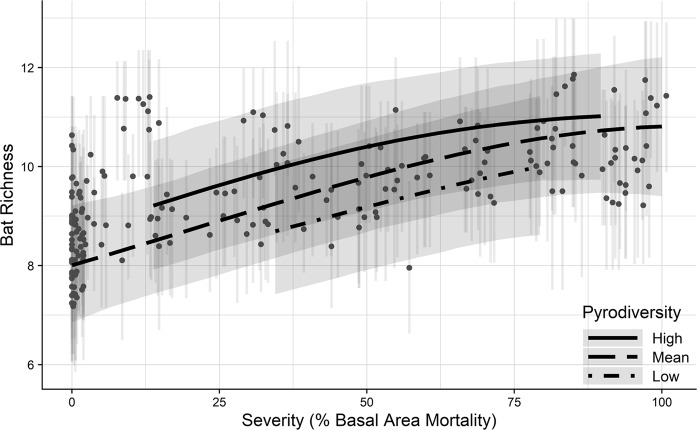
Model predicted species richness across the full range of burn severity (percent mean basal area mortality). Low, mean and high of pyrodiversity represent −2, 0, and 2 standard deviations from the mean, respectively. Mean predicted richness of each survey period is displayed as points, which are slightly offset using a random “jitter” to display overlapping values. Error bars represent 90% credible intervals. Richness predictions are made using mean severity calculated with a 250 m radius. Because there is little variation in burn severity near extremes (Figure S1), our predictions for high and low pyrodiversity are limited to where sampled pyrodiversity diverged at least 2 standard deviations from the mean.

Aggregated model predictions show increasing bat richness with increasing burn severity and with pyrodiversity. On the low end, an average of 8.0 species (90% CI: 6.9–9.2) are expected to be present in unburned (0% mortality) areas. Species richness is expected to maximize at approximately 11.0 species (90% CI: 9.7–12.3) with moderate to high basal area mortality and high pyrodiversity in the surrounding area (Fig. 6).

Jaccard’s pairwise dissimilarity was positively correlated with severity distance (r = 0.24). The greatest dissimilarity among severity groups was between unburned points and high-severity (>75% basal area mortality) points where 42% of the species differ on average. Dissimilarity was smallest within the high-severity group where an average of 31% of species differ between points, while dissimilarity was 36–37% within other severity groups (Fig. S3).

## Discussion

Bats respond to wildfire in the Sierra Nevada Mountains in varied but often positive ways. Site occupancy by at least 6 species (35% of the community) increased and one species likely decreased with burn severity. However, species are predicted to maximize occupancy rates at various levels of severity (Figs 3 and 4). The effect of pyrodiversity (variation in burn severity) was less consistent, with at least three species showing a positive and two showing a negative relationship with the measure (Figs 3 and 5). At the community level, models predict species richness to increase with both burn severity and pyrodiversity. Because variation in burn severity is low at the extremes of fire effects (i.e. 0 and 100% basal area mortality; Figure S1), the most speciose areas are likely those with moderate- to high-severity and high pyrodiversity, with the lowest number of species occurring in unburned areas (Fig. 6). Mean community dissimilarity was greatest between unburned and high-severity sites, with dissimilarity showing a positive correlation with the magnitude of severity difference (Figure S4). Taken together, these findings suggest bats benefit from wildfire, including high-severity wildfire, and that a heterogeneous mix of burn severities would be most beneficial to bat diversity in Sierra Nevada forests.

Forest disturbances can reduce clutter and increase accessibility of foraging and commuting habitats for some species. This has been most rigorously tested for timber harvest treatments where bat activity and/or occupancy is often greater for clutter-intolerant species in thinned stands^6,40–42^. In contrast, assessments of fire severity on bats are more limited in number and ability to infer species-level effects. A concurrent study conducted in northern Sierra Nevada forests found bat functional traits associated with open areas were positively correlated with high-severity fire and fire frequency, whereas traits associated with habitat clutter tolerance were negatively correlated with such fire characteristics^7^. Similarly, a previous study in southern Sierra Nevada mixed conifer forests found higher activity rates of small-bodied species within high-severity areas^8^, and a study in Idaho forests found that overall bat activity was higher in high-severity areas than unburned areas^43^. Studies of prescribed fires that mimic low- to moderate-severity wildfire have shown slightly positive to neutral response of bat species or groups^42,44,45^. Our results showing positive relationships between occupancy and burn severity for many species may indicate that foraging and/or commuting rates are increased by wildfire. A minority of species are predicted to occur most often in low-moderate severity or unburned forest (i.e. canyon bat and small-footed myotis; Fig. 4) and would be negatively affected by high-severity wildfire. These results provide evidence that forests with a long-term lack of disturbance are suboptimal habitat generally. Indeed forest densification as a result of fire suppression may have degraded bat habitat to such an extent that even uncharacteristically severe wildfires may improve habitat quality for the community if burning in areas with a deficit of wildfire.

Wildfire is an important driver of biodiversity, with the magnitude and direction of effects varying among taxa. Here, bat richness is predicted to rise with mean burn severity, although within-class beta diversity was lowest among high-severity points (Fig. 5 and S3b). An analysis of Sierra Nevada birds found an approximately equal number of species reaching maximum density across the severity gradient^28^. Plant community richness may be maximized in low- to moderate-severities in mixed-conifer forests of California^46,47^. Lichen diversity is sensitive to wildfire, with significant declines in richness with increasing severity^16^. Vagile species such as bats and birds likely respond to habitat composition and structure at broader scales than sessile organisms, which may partially explain differences among taxa. Indeed bat occupancy models using severity data calculated at a relatively broad scale (i.e. 500 m radius) were often selected during model comparison (Table S1). Disparate responses to burn severity among taxa have important implications for the maintenance of biodiversity in fire-prone systems, and the mechanisms of these differences deserve further exploration.

Beyond mean burn severity, pyrodiversity may increase biodiversity by diversifying available ecological niches^5^. We found some evidence of a positive relationship between pyrodiversity and bat diversity with approximately one additional species (6% of the community) predicted to occur on average when moving from areas of low to high pyrodiversity (Fig. 6), and greater community dissimilarity between points with increasingly different burn severities (Figure S3a). A similar pattern was observed for Sierra Nevada bird and plant-pollinator communities^33,48^, suggesting a positive relationships between pyrodiversity and biodiversity may be generalizable within the region. However, the strength of such a relationship likely varies among biomes, fire regimes, and across environmental gradients^49^. Among individual species, we observed positive relationships with pyrodiversity for Yuma myotis, fringed myotis and California myotis, but negative associations for pallid bat and big brown bat (Figs 3 and 5). While pyrodiversity is typically considered to promote biodiversity by accommodating species of differing specialties, it may also benefit individual species that require resources available in contrasting severities^50^, or those that forage along habitat edges. Conversely, pyrodiversity may be detrimental to habitat specialists, and a landscape that maintains distinct habitat patches within a matrix of burn severities may be needed to accommodate some species.

Forest management in fire-prone landscapes is increasingly focused on reducing the risk of large patches of high-severity wildfire by reducing stand densities and fuel loads. Preventative measures include use of natural wildfires through managed wildfire programs^51^, or fire surrogates such as prescribed fires and mechanical fuel reduction treatments^52^. Managed wildfires likely benefit forest bats by both decreasing clutter as well as creating post-fire resource pulses such as abundant aquatic insect prey^43^ and roost habitat in the form of damaged or killed trees. This may also be true of prescribed fires, but to a lesser degree because such treatments are designed to limit high-severity fire. Mechanical fuel reduction treatments also reduce clutter and theoretically could improve foraging habitat for some species. However, in contrast to fire, these activities can negatively impact roost availability for tree- and snag-roosting species^42^. As management of western forests in an age of changing fire regimes increasingly emphasizes fire and fire-surrogate activities, and as bats face compounding threats such as white-nose syndrome^20^, effective bat management will necessitate an understanding of how these disturbances affect the multiple forest resources bats require.

While we successfully modeled the full Sierra Nevada bat community, model estimates of occupancy rates and/or fire effects remain highly uncertain for a number of species. Such lingering uncertainty can be attributed to a combination of natural rarity of such species, challenges to recording and classifying their echolocations, or both. As acoustic technology, classification algorithms, and statistical methods continue to improve, these limitations will likely be reduced in the future. Additionally, future work should build on the findings presented here by exploring the relative importance of fire effects on foraging clutter, roost availability in the form of snag creation, and alterations to prey communities^43^. Habitat resources and bat occupancy also likely change with post-fire succession^7^ and a more complete understanding of these temporal dynamics would improve our ability to conserve bats across landscapes with complex fire histories.

## Conclusions

Prior to euromerican settlement, western dry forests experienced frequent wildfire which created open stands of large fire-resistant trees, interspersed by patches of both early and late successional habitats^11^. However, following a century of fire suppression, much of Sierra Nevada forests have experienced an absence of wildfire, and have become denser, and more dominated by small trees^11,12^. For bat species that require uncluttered forests for foraging, and/or large live and dead trees for roosting, this transition may have been detrimental. Here we demonstrate increasing rates of bat occupancy and diversity when fires do occur, with the greatest benefit predicted in relatively severe but pyrodiverse areas. Thus, observed increases in burned area and average burn severity in recent decades may benefit the forest bat community generally, especially where it replaces uncharacteristically dense, fire-suppressed habitats. However, this benefit may be limited where increasingly large fires create homogenous high-severity patches rather than a diverse mosaic of burn severities.

## Supplementary information

Supplementary Material

Dataset 1

Dataset 2

Dataset 3

## Data Availability

All data used in analysis as well as Stan model code are included as Supplementary Material to the article. Additional supporting R code can be found at https://github.com/zacksteel/FireBats.
