# Supplementary material for: The effects of wildfire severity and pyrodiversity on bat occupancy and diversity in fire-suppressed forests
Source: Sci Rep. 2019 Dec 5;9:16300. doi: 10.1038/s41598-019-52875-2 (PMC6895131; doi:10.1038/s41598-019-52875-2)
Supplement: Supplementary file 1 — Supplementary Material [file 41598_2019_52875_MOESM1_ESM.pdf]

The effects of wildfire severity and pyrodiversity on bat occupancy and diversity in fire-suppressed forests

Steel Z. L., Campos B., Frick W.F., Burnett R., and Safford H. D.

Supplementary Table S.1. The common name, scientific name and select species characteristics for 17 species known to occur in the Sierra Nevada, ordered by ascending body mass within family. The species characteristics include mean values for body mass (Jones et al. 2009), wing-loading (Norberg and Rayner 1987), call frequency at maximum power (FME), and call duration (Szewczak 2010).

Common Name	Scientific Name	Mass (g)	Wing-loading (kg/m^2)	FME (kHz)	Call Duration (ms)	Selected Scale
Vespertilionidae						
Canyon bat	*Parastrellus hesperus*	3.5	6.9	48.3	5.5	500
California myotis	*Myotis californicus*	4.4	4.8	52.8	3.8	250
Small-footed myotis	*Myotis ciliolabrum*	4.9	6.7	49.1	3.2	50
Yuma myotis	*Myotis yumanensis*	5.2	7.8	55.2	5.5	250
Long-eared myotis	*Myotis evotis*	6.9	6.1	39.1	3.7	250
Little brown bat	*Myotis lucifugus*	7.8	7.5	44.5	6.0	100
Fringed myotis	*Myotis thysanodes*	8.5	6.2	30.7	3.9	500
Long-legged myotis	*Myotis volans*	8.6	8.3	48.0	4.8	500
Townsend's big-eared bat	*Corynorhinus townsendii*	10.3	7.2	31.1	4.6	50
Silver-haired bat	*Lasionycteris noctivagans*	10.9	8.2	28.8	9.2	50
Spotted bat	*Euderma maculatum*	16.2	--[a]	12.5	3.2	500
Big brown bat	*Eptesicus fuscus*	17.3	9.4	31.9	7.8	500
Western red bat	*Lasiurus blossevillii*	12.3	14.0	14.6	10.7	250
Pallid bat	*Antrozous pallidus*	22.2	8.1	31.0	6.8	500
Hoary bat	*Lasiurus cinereus*	26.8	16.5	20.8	11.0	500
Molossidae						
Mexican free-tailed bat	*Tadarida brasiliensis*	12.2	11.5	28.0	11.5	500
Western mastiff bat	*Eumops perotis*	50.9	14.0	41.6	10.7	250

[a] Wing-loading not documented by Norberg and Rayner (1987).

Supplementary Figure S.1. The relationship between mean and the standard deviation of basal area mortality at four spatial scales, as defined by 50m, 100m, 250, and 500m radii from sample points. A slight horizontal jitter is added to display overlapping values of mean basal area mortality.

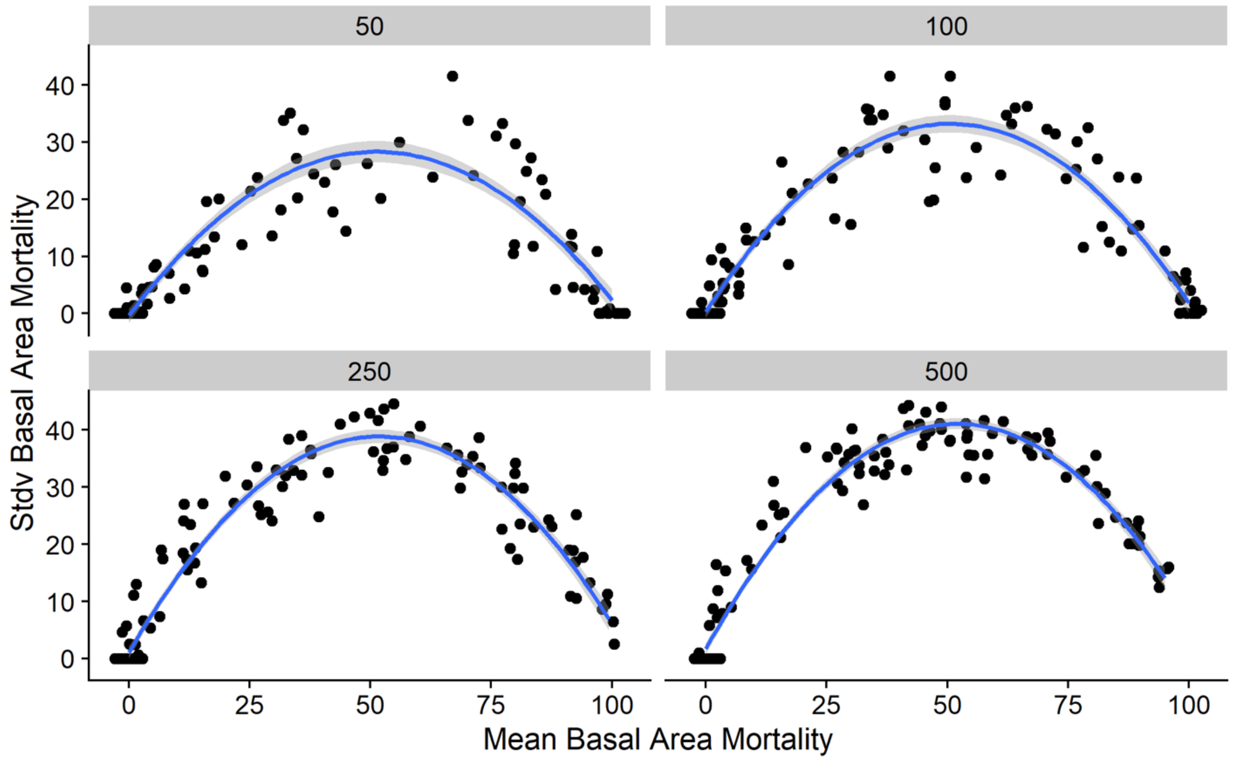

Supplementary Figure S.2. LOOIC model weights by species and scale. Selected models are listed in Table S.1.

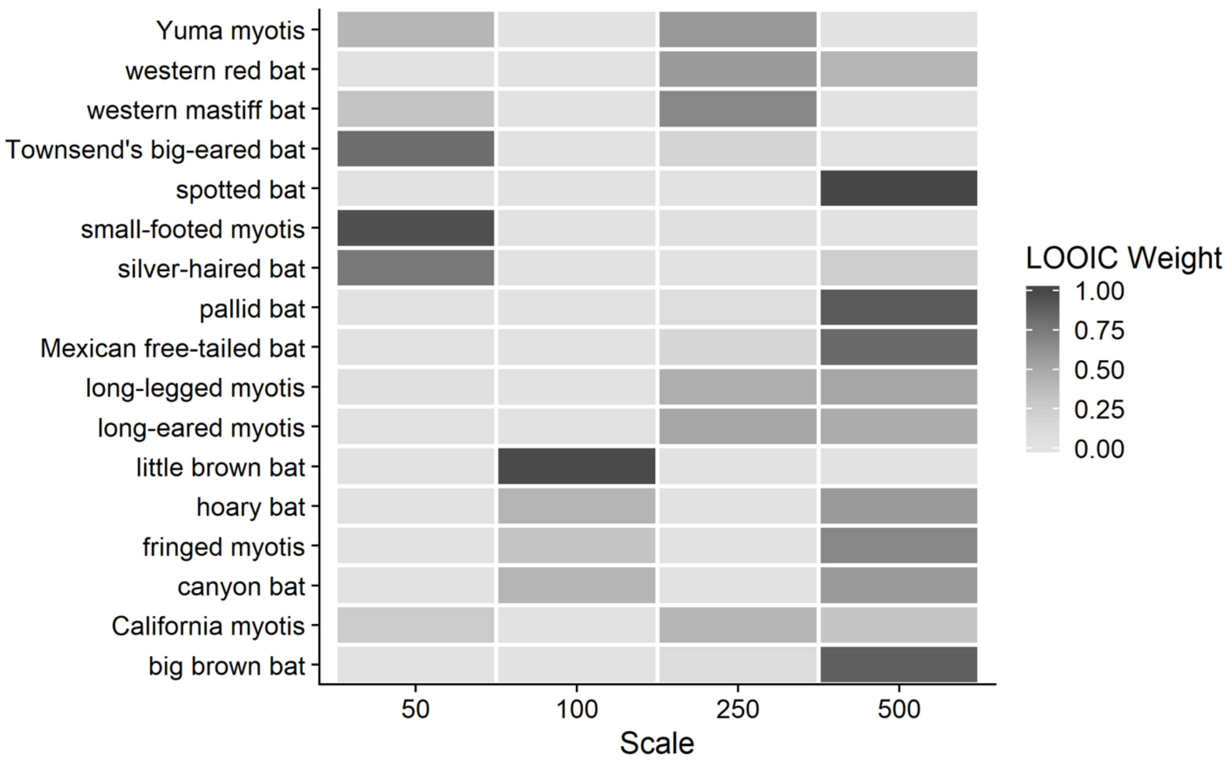

Supplementary Figure S.3. Jaccard's dissimilarity across burn severity (basal area mortality BAM). a) Pairwise dissimilarity distributed by severity distance (i.e. difference in BAM), and b) mean dissimilarity between severity groups. The magnitude of dissimilarity is displayed both by color and circle size in the group-wise comparison. Severity groups are defined as unchanged (U; 0% BAM), low (L; 1-25% BAM), moderate (M; 25-75% BAM), and high (H; >75% BAM). Comparisons are made using mean severity calculated with a 500m radius (Figure S.1).

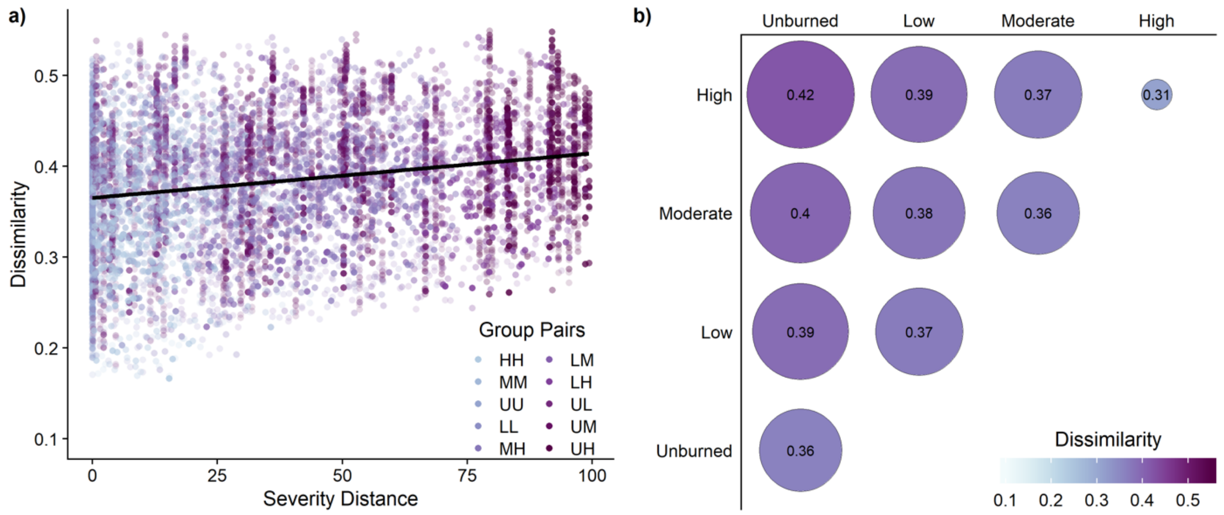

Supplementary Data S.1. Bat occurrence and environmental data used to fit occupancy models.

Supplementary Data S.2. Parameter estimates for bat species models. The mean, 90% credible interval and probability an effect is positive (i.e. the proportion of the parameter posterior distribution above zero) are listed for each parameter.

Supplementary Code S.1. Occupancy model code.

Literature Cited in Supplementary Material

Jones, K. E. *et al*. PanTHERIA: a species-level database of life history, ecology, and geography of extant and recently extinct mammals. *Ecology* 90:2648–2648 (2009).

Norberg, U. M., & J. M. V. Rayner. Ecological morphology and flight in bats (Mammalia; Chiroptera): wing adaptations, flight performance, foraging strategy and echolocation. *Philosophical Transactions of the Royal Society B: Biological Sciences* 316:335–427 (1987).

Szewczak, J. M. SonoBat version 3.2.1. (2010). www.sonobat.com.
